# Population Abundance and Density Estimates of Poorly Documented Near-Threatened Calabar Angwantibo (*Arctocebus calabarensis*) in Oban Hills Region

**DOI:** 10.3390/ani14091374

**Published:** 2024-05-02

**Authors:** James Kehinde Omifolaji, Sunday Opeyemi Adedoyin, Emmanuel Tersea Ikyaagba, Tauheed Ullah Khan, Victor Abiodun Ojo, Yiming Hu, Abideen Abiodun Alarape, Saka Oladunni Jimoh, Huijian Hu

**Affiliations:** 1 Guangdong Key Laboratory of Animal Conservation and Resource Utilization, Guangdong Public Laboratory of Wild Animal Conservation and Utilization, Institute of Zoology, Guangdong Academy of Sciences, Guangzhou 510260, China; 2 Department of Forestry and Wildlife Management, Federal University Dutse, Dutse 720222, Jigawa State, Nigeria; 3 Department of Forestry and Wildlife Management, Prince Abubakar Audu University, Anyigba 272102, Kogi State, Nigeria; 4 Department of Social and Environmental Forestry, University of Agriculture, Makurdi 970001, Benue State, Nigeria; 5 Department of Forestry and Wildlife Management, University of Maiduguri, Maiduguri 600230, Borno State, Nigeria; 6 Department of Wildlife and Ecotourism Management, University of Ibadan, Ibadan 200132, Oyo Sate, Nigeria; 7 Department of Social and Environmental Forestry, University of Ibadan, Ibadan 200132, Oyo Sate, Nigeria

**Keywords:** arboreal mammals, Calabar angwantibo, near-threatened species, nocturnal primates, loris, density, encounter rate, population monitoring

## Abstract

**Simple Summary:**

The Calabar angwantibo is a mammal species found only in southeastern Nigeria and southwestern Cameroon. This study tried to assess the abundance and density of this species in the Oban Hills Region of Nigeria. Our results showed that the species has an estimated population of 4456 individuals with an estimated density of 1.56 animals/km^2^ in the study area. Our research underlines the significance of species monitoring and provides valuable insight into the Calabar angwantibo’s population in rainforest ecosystem of Oban Hills Region.

**Abstract:**

Population abundance and density estimates play important roles in biodiversity conservation assessment and can lead to prioritization of conservation efforts, strategies, and management. The Calabar angwantibo (*Arctocebus calabarensis*) is a poorly studied, Near-Threatened nocturnal, arboreal mammal species occurring only in the lowland moist tropical rainforest blocks of southeastern Nigeria and southwestern Cameroon. Like other arboreal nocturnal mammals, there are gaps in knowledge of the distribution and abundance of this species, which may be facing population declines due to habitat loss and hunting. In this study, we investigated the abundance and density of *A. calabarensis* in the Oban Hills Region of Nigeria. We conducted systematic distance sampling survey along 32 transects in different habitats in a 1227 km of survey efforts. A total of 41 sightings of *A. calabarensis* were detected, resulting in an abundance of 7345 individuals (95% CI = 1.51–4.37) with an estimated density of 2.57 animals/km^2^. The estimated population abundance is 6515 individuals in closed-canopy forests and 830 individuals in secondary forests, with encounter rates of 0.52 individual/km^2^ and 0.60 individuals/km^2^ in the closed canopy and secondary forest habitats. The global estimates encounter rate of *A. calabarensis* across the habitat types is 0.33 individuals/km^2^ and population abundance of 4456 individuals. Our findings indicate that the *A. calabarensis* populations can adapt to low changes in forest habitat modifications resulting from increasing and widespread forest disturbance by human-dominated activity, which is giving way to forest clearance for agriculture cultivation and infrastructural development. Our findings help to fill a knowledge gap regarding this species and may help establish a baseline for future management, population monitoring, and conservation of the cryptic population of *A. calabarensis* in Cross-Sanaga Forests.

## 1. Introduction

African rainforest ecosystems are characterized by a high diversity of primates, including nocturnal arboreal mammals [1,2,3,4,5]. In central and western Africa, it is not uncommon for seven to eight primate species to occur in sympatry [2,6]. The Oban Hills Region is no exception and also represents crucial biodiversity hotspots and habitats of significant conservation value in Cross-Sanaga forests [7], particularly for primates; this region contains one-quarter of all the primate species that occur widely throughout the Guineo-Congolian forest [2,6,8,9,10]. However, deforestation and human exploitation of these primates for bushmeat have drastically affected their populations, including those in protected areas [11,12,13,14,15]. Moreover, since these taxa are primarily restricted to tropical rainforest ecosystems, 53% of all primate species are now classified as threatened with extinction by the IUCN Red List of Threatened Species [16]. Consequently, effective strategies for conservation planning are urgently required to ensure species’ long-term survival. Obtaining accurate population density, abundance, and distribution estimates of poorly studied and threatened taxa across a range of habitats are vital prerequisites for informing such strategies and subsequent on-the-ground actions [17,18]. Under these circumstances, basic information on primate ecology has become increasingly important. In particular, population density is valuable for assessing population status, monitoring population density changes, and identifying priority areas for conservation.

Surveying arboreal nocturnal animals in tropical forest ecosystems to obtain reliable information such as density is difficult. This is because these animals are cryptic, small, and often found in the dense tree canopy [19] and lianas, with some species occurring at a height of 15 m or more in low, incredibly dense shrubs, making it difficult to detect them at night [20]. Similarly, censusing animals at night can be demanding for investigators in a dense and moist tropical forest ecosystem [21,22]. Calabar angwantibos are primarily nocturnal, cryptic arboreal-dwelling mammals [5,23,24]; identifying this species in the forest can be challenging, which makes it difficult to collect important data for population monitoring and trends. Therefore, it is essential to conduct extensive studies on their distribution, behavior, and ecology to strengthen population monitoring efforts of this species [5]. This makes it difficult to establish a baseline understanding of Calabar angwantibo ecology and distribution in West and Central Africa.

Calabar angwantibos (*Arctocebus calabarensis*) are arboreal nocturnal primates of the order Lorisidae endemic to sub-Saharan Africa. Only recently assessed and classified as Near-Threatened by the IUCN Red List, the species has been recognized as being at extinction risk due to habitat loss and the bushmeat trade [19,25,26,27]. Calabar angwantibos have overlapping geographical ranges with bushbabies and pottos in west and central African tropical rainforests, though they have distinct habitat preferences when found sympatrically with other bushbabies [19,20,28,29]. The Calabar angwantibo is a relatively understudied species, and its population is believed to be declining due to habitat loss and hunting [15,19]. The species can utilize dense forest canopies, but are mainly understory specialists, and are known to occur in areas with high liana density; their conservation is closely tied to the protection and preservation of rainforest ecosystems. The species is susceptible to forest clearance and conversion to agricultural land and tends to adapt to secondary forests with undergrowth [19,24]. Published studies documented the existence of arboreal forest-dwelling nocturnal strepsirrhines in southern Nigeria, including angwantibos, galagos, and pottos [5,8,23,24,30,31,32,33].

In light of the vulnerable conservation status of the angwantibos and the current lack of information, threats of habitat loss, and hunting for bushmeat trade of this highly cryptic nocturnal primate, it is critical that surveys are undertaken to ascertain the population density of Calabar angwantibos [19]. Until now, the few published studies that have provided information on distribution and frequency of observation were systematically conducted outside the current sites and mainly based on short-term surveys where other nocturnal primates were particularly abundant [5,23,30,34,35,36,37]. These studies have no indications of how common or rare the animals are. This suggests that angwantibos were sporadic to sight but rare at other sites. Here, we aim to investigate the population of *A. calabarensis* in the Oban sector of Cross River National Park, Nigeria to assess its abundance and distribution. Specifically, our objectives were to (1) record sightings of *A. calabarensis* and (2) estimate the population density of *A. calabarensis* in Oban Hills Forest.

## 2. Materials and Methods

### 2.1. Study Area

**Figure 1:**
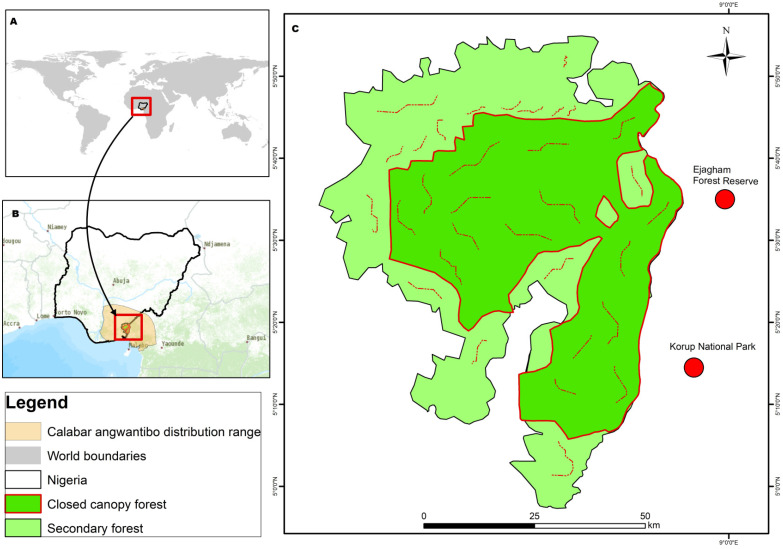
Map of Oban Hills Region of Cross River National Park, Southeastern Nigeria. (**A**) global distribution range of Calabar angwantibo; (**B**) Calabar angwantibo range in Nigeria and Cameroon, (**C**) showing the map of the study location.

We conducted our study in the Oban Hills Sector of Cross River National Park (CRNP) (5°15′ N 25′ N 8°30′45′ E, Figure 1), divided into two ranges: Oban East and Oban West. This is a UNESCO World Heritage site, Important Bird Area, and Biodiversity hotspot [38] (Myers et al., 2000).

It is the most important tropical rainforest ecosystem in Nigeria as well a carbon sink at an elevation ranging from 100 m in the river valley to 1000 m above sea level in the mountainous area [38,39,40]. The Oban Forest, CRNP is contiguous with Ejagham Forest reserve and Korup National Park, Southwest Cameroon, forming a single protected ecological zone in Central and West Africa [8,14,41]. It is the most extensive tropical forest protected area, with 3000 km^2^ of lowland tropical rainforest ecosystem. It is home to sixteen (78%) primate species found in Nigeria, including the Endangered Nigeria–Cameroon chimpanzee (*Pan troglodytes ellioti*), Critically Endangered Preuss’s red colobus (*Piliocolobus preussi*), crowned guenon (*Cercopithecus pogonias*), and drill (*Mandrillus leucopheaus*) [10,42], and other endangered animals such as African forest elephants (*Loxodonta cyclotis)* and the white-bellied pangolin (*Phataginus triscupis*). It is regarded as the last stronghold of tropical rainforest in Nigeria [8] and also provides a suitable habitat for a diverse range of fauna species [8], over 900 butterflies [43,44], an Important Bird Area (IBA) with over 350 bird species [45,46]. It is home to 1568 plant species, including 1303 flowering plants, 141 lichens, and 56 moss species, of which 77 are endemic to Nigeria [39]. The typical tree species include bois de rose (*Berlinia confuse),* African walnut *(Coula edulis),* uguekpokin (*Hannoa klaineana),* bush mango *(Klainedoxa gabonensis),* African mahogany (*Khaya grandifolia*), and red ironwood (*Lophira alata*). The annual rainfall is 3000 mm in the southern parts and 2500 mm in hilly and mountainous areas, and the climate is tropical and humid [47]. The temperature is 25 °C to 27 °C in January, but it usually rises slightly above 30 °C in July. Relative humidity is about 75 to 95% in January, but it reduces gradually during the harmattan period, which runs from November to February, characterized dry north-easterly trade wind from the Sahara Desert that conveys fair and dusty weather to the region [48,49].

### 2.2. Data Collection

We conducted this study as part of the biological component of a regional wider research project investigating impact of land use types on biodiversity conservation in the Oban Hills Region. The Oban Hills Region landscape of the CRNP was surveyed on a bi-monthly basis between February 2010 and March 2014 for the Calabar angwantibos census in Oban Hills Region. The area was categorized into two habitat types based on intensity of human disturbance activity around the park, namely closed-canopy and secondary forest. The broad categories of land use type were: (1) closed-canopy forest (>75% canopy cover), which comprises matured, closed-canopy forest with numerous arboreal pathways and scarce forest undergrowth; and secondary forest (>45% canopy cover), which comprises a mixture of regenerating forest and dense shrub vegetation, as described [39,50]. The transects were laid in different habitats to avoid bias associated with data collection on object of interest (animals) due to different levels of anthropogenic impacts. As described by [51] Buckland, 2001, the transects survey was employed for this study. Several researchers have employed this method to determine animal species and abundance, especially in tropical rainforest ecosystems, due to the nature of mammalian species and the area’s topography.

In each land-use type, 16-line transects (2 km length each) were established with the aid of a global positioning system (GPS), considering the landscape topography based on a stratified sampling technique. An equal number of transects were located in closed-canopy forest (core zone: 361 km^2^) and secondary forest (buffer zone: 367 km^2^), and sufficiently placed at an interval of 600 m apart from two neighboring transects [52,53]. Line-transects was the main technique employed to investigate mammals, especially nocturnal arboreal species in the tropical rainforest ecosystem; due to the dense nature of forest cover and the intensity of anthropogenic activities, we ensure that the starting points of all transects begin from each land-use type of the forests. The survey was conducted by walking along each transect between 19:30–23:00 h GMT while looking ahead and laterally to the direction of travel to detect and identify Calabar angwantibos and other nocturnal species with the aid of a headlight. Each transect was walked by trained observers within a radius of 50 m on both sides of the line transects. Within all the land-use types, we focused on concentrated searches in the tree branches, forest canopy covers and lianas once we observed the presence of Calabar angwantibos. We followed transects and maintained a straight line to have little or no influence on perpendicular estimation with the aid of a Nikon Laser rangefinder. We conducted intensive searches in all habitat types for Calabar angwantibos, and the following data were recorded: species, number of individuals, location, time, vocalization, signs of anthropogenic activities, sighting distance, and perpendicular distance from the transects. In addition, we categorized perpendicular distance data into five groups: 0–5 m, 6–10 m, 11–15 m, 16–20 m, and 21–25 m across all habitat types. All surveys were conducted by trained observers familiar with distance estimation. All detections were estimated within 20 m from the centerline, a natural truncation of distance created by the limitations of our headlights. During nocturnal surveys, we used adjusted headlights with red light filters that do not disturb the nocturnal animals and minimize interference, as the animals cannot see red light, and therefore allowed us to observe their activity as outlined [54,55]. All detections were made visually, either by detecting the *A. calabarensis* reflective *Tapetum lucidum* or when an animal directly crossed over the path. No vocalizations were heard or recorded during the entire study period. During the study, a total of 1227 km of survey effort were accumulated during the nocturnal sampling of Calabar angwantibo in the whole study site.

### 2.3. Data Analysis

To obtain a baseline estimate of Calabar angwantibos density, we performed distance sampling analyses on each habitat type as outlined in [51,53] conducted in the computer software program DISTANCE 7.2 [52,53]. We estimated detection probability and proportion of animals detected in the region by fitting a detection function to the observed data and modeled using recorded distance of the animals from the line [53]. Habitat types were used as a covariate in modeling detection probability for the species sighted during the survey, and a correct detection probability was generated using pooled distance data from all strata, thus avoiding the need to accumulate the normally recommended 60–80 observations. We calculated Calabar angwantibo density, encounter rate, and abundance in each habitat type and whole habitat cover in the area. The detection function was modelled using the half-normal and hazard-rate keys, which were fitted to pool data from each habitat and all study sites combined and from all habitats (and all sites). The half-normal key with cosine adjustments was selected for the survey, whereas hazard-rate keys with cosine adjustments were selected for all remaining analyses. In addition, we used a histogram to evaluate indications of potential detection distance further away from the line. Using the truncation distance, we assessed the data fit to several detection function models recommended [52]: uniform key with cosine adjustment; half-normal key with cosine adjustment; hazard-rate key cosine adjustment. We selected the best-fit model based on the Akaike Information Criterion (AIC), where ΔAIC values were <2.00. Among each best-fit detection function model, we chose a final model based on 3 goodness-of-fit tests (Kolmogorov–Smirnov (K-S), Cramer–von Mises (CvM unif), and Cramer–von Mises (CvM cos)) were *p* > 0.05. Due to the visible drop in detection probability near the zero line in most datasets, distance was grouped into 8–10 m distance classes to obtain a good fit. Some right truncation (5–10% of observations) was applied to all data sets.

## 3. Results

The nocturnal sampling study findings reveal a total of 41 *Arctocebus calabarensis* were recorded in closed-canopy and secondary forest; detections made on the transects lines varied in the habitat types survey in study sites. The result reveals that *A. calabarensis* distribution slightly differed in the two habitat types, and a total number of 19 sightings were recorded after total survey efforts of 361 km in the closed canopy forest and 22 sightings in the secondary forest with 367 km survey efforts, respectively. The overall density estimates of *A. calabarensis* in Oban Hills Region are 1.56 individuals per km^2^, with an estimated abundance of 4456 individuals (95% = 0.97–2.51). The Akaike information criterion (AIC) recorded ranged between 97.36 and 118.05 for closed-canopy and secondary forest habitat types. The encounter rate for the all-habitat types is 0.33 individual/ km^2^, and the difference in AIC for the models was very small and close to density estimates. The density and population abundance calculated are 3.15 km^2^, 6515 individuals (95% CI = 1.75–5.69) for closed-canopy, and 2.26 km^2^, with a population abundance of 830 individuals (95% CI = 1.20–4.26) for secondary forest, respectively. Over the course of the distance sampling survey, the encounter rate ranged between 0.52–0.63 angwantibo/km^2^ (Table 1). Calabar angwantibos detection probability varied between the habitat types: secondary forest (*p* = 0.94) and closed-canopy forest (*p* = 0.59).

**Figure 2:**
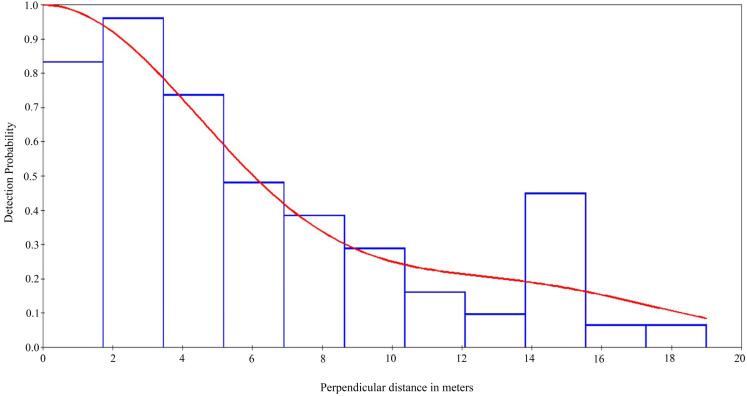
Histogram indicating perpendicular distance to the transects and detection probability of sighting Calabar angwantibo in Oban Hills Region, CRNP.

The results reveal only slight differences in population density estimation between the two habitat censuses. Our findings indicate that the perpendicular distance estimation of *A. calabarensis* ranged from 1 m to 15 m for most individuals sighted to the observers. The proportion of species sighting is higher in the secondary forest habitat, and the average sighting perpendicular distance ranged between 3.8 m–6.5 m for the species across the habitat types. Our results indicate that the detection rate of *A. calabarensis* species population in the Oban Hills Region decreases from 1.0–0.2 with increasing perpendicular distance from the transects line. *A. calabarensis* species population density differs across the land-use types; the detection was plotted and superimposed on the histogram, showing the detection probability decreasing further from the transects line to the *A. calabarensis* detected (Figure 2). The observed average height recorded for trees and lianas used by Calabar angwantibo ranged from 2 m–4 m. Our findings reveal that the species were mainly solitary individuals and patchily distributed in some locations across the region.

## 4. Discussion

In summary, our findings provide a significant baseline for population density estimates of Calabar angwantibos (*Arctocebus calabarensis*) across different habitat types; however, another study should urgently be conducted to see if there any changes to the species population within Oban Hills Region, Cross River National Park, one of the protected area sites for the conservation of this IUCN Red List of Threatened Species: Near Threatened, endemic and poorly studied lorisids in Cross-Sanaga Area. Our data suggest that the abundance of Calabar angwantibos differs across the habitat types in the Oban Hills Region of Cross River National Park, although 41 individuals of Calabar angwantibos were recorded in the two habitat types of closed-canopy and secondary forests in the Oban Hills Region of CRNP. This number is a more than 100 percent increase, higher than the combined figure recorded for the five forest forests (Iko Esai, Itu, Wilberforce Island, and Tombia forests) from a previous study in southeastern Nigeria [5] and Southwest Cameroon that varied between one and five individuals [36]. These findings are very important because they fill the gap by providing novel insight into the ecology and abundance of the Calabar angwantibos population in the lowland moist tropical rainforest, which is facing population declining trends. Yet, these findings represent a significant step towards providing a baseline for a species that has largely hitherto only been recorded as present or absent in the region by addressing population monitoring gaps, a departure from previous population studies in the area that relied on casual observations of this vulnerable cryptic arboreal species. The majority of sightings of this species in the past have been in forest habitat type; however, our sighting recorded a few more individuals in the secondary forest. The higher encounter rates we observed in the closed canopy forest are consistent with habitat association preference of African nocturnal primates. This preference may be attributed to availability of abundant food and shelter resources in the lowland moist rainforest for the African primates [56]. The diverse and rich vegetation cover types within and around Oban Hills Region provides suitable habitats for the species, supporting the species with specific habitat needs.

Thus, our study fills a significant knowledge gap on *A. calabarensis* population density globally and within this region. In comparison, some efforts focused on species documentation in Southeastern Nigeria and southwestern Cameroon [2,5,19,24,34]. To our knowledge, non-systematic encounter of *A. calabarensis* population density nocturnal survey study has been investigated across the different habitats of Oban Hills Regions, Nigeria. In the literature, density estimates for *A. calabarensis* vary according to the sampling/analytical methods [24,36]—our overall estimated density was 2.57 individuals per km^2^ within the Oban Hills Region study site. This study’s encounter rate of 0.5 km^2^ recorded is lower than the estimated encountered rate of 0.7 km^2^ reported in the same region of southeastern Nigeria [5,24]. However, the population encounter rate obtained in this study, 0.53 km^2^, is higher than the 0.16 km^2^ individual in southwest Cameroon and sporadic detection reported in four locations west of River Cross, southern Nigeria [5,35,36,37]. This disparity may be due to a systematic survey of the Calabar angwantibo population in the current study, unlike the previously published studies where the species is the least recorded among nocturnal primate surveys estimate density and casual observation.

Our findings corroborate [23] that the populations *A. calabarensis* can adapt to low changes in forest habitat modifications resulting from increasing and widespread forest disturbance by human-dominated activity, which is giving way to forest clearance for agriculture cultivation and infrastructural development as well as the recent appearance in bushmeat reports [15,23,37,57]. Furthermore, Nigeria has witnessed human population growth and economic development intensification, leading to an estimated loss of 59.4% forest cover between 1990 and 2015 and causing wildlife population decline from habitat loss and increasing hunting pressure on wildlife (small nocturnal primates), including Calabar angwantibo [15,19,20,37,57,58,59,60,61,62].

The distribution and density of Calabar angwantibo in the habitat types surveyed were differs in closed canopy forests and secondary forests. We observed that *A. calabarensis* occurred in two habitat landscapes of closed canopy and secondary forest, which is synonymous with species diversity and richness for terrestrial mammals in the Oban Hills Region [8,33]. Equatorial countries with significant topographic relief and moist forest cover have been observed to play a key role in global primates’ hotspots, diversity, richness, and endemism for arboreal mammals [7,8,19,26,27,33,63] as evidenced in Africa, Asia, and Central and South America. These overall findings remain significantly similar in the closed-canopy and secondary forest habitat types; this finding confirmed and supported the discussion that *A. calabarensis* is highly susceptible to human-dominated activities and habitat loss, a common phenomenon of habitat loss. Our findings during surveys reveal that Calabar angwantibos favor lianas and were specifically found close to small scale forest clearances in the secondary forest of the park boundary. This study corroborates those previously published studies [19] that found that, despite deforestation and the bushmeat hunting trade, the species is still restricted to closed-canopy and secondary forests with undergrowth lianas as previously documented [23,24,30].

*Arctocebus calabarensis* were found to have a preference for understory closed-canopy and secondary rainforest, low undergrowth with abundant liana and vines below 5 m for navigation [19,24,28], which is consistent with our result of increasing density and population distribution within closed-canopy and secondary forest habitats. The terrain of primary rainforest ecosystems of the protected area is also documented as a central sanctuary of *A. calabarensis* habitats preference particularly along forest edges [19,23,24], which is relevant given that this habitat is the most secured and contiguous area for the conservation of *A. calabarensis* in Nigeria and Cameroon. Our findings corroborate those previous studies in the same area [5] that close canopy forest habitats support more nocturnal primates, including Calabar angwantibo, than the areas subjected to agricultural cultivation and anthropogenic disturbances. Despite Calabar angwantibo’s nocturnal behavior, human-dominated activities negatively influence the region’s nocturnal arboreal mammals’ distribution and density.

## 5. Conclusions

In conclusion, this study expands our knowledge about the poorly studied cryptic nocturnal *A. calabarensis* of great interest for conservation in West and Central Africa tropical rainforest biomes. Calabar angwantibo population estimate indicates 2.57 individuals/km^2^ for the habitats in the region. Furthermore, we found a density estimate of 3.15 individual per km^2^ in closed-canopy forest and 2.26 individuals per km^2^ in secondary forest. Meanwhile, the encounter rate was 0.5 individuals/km^2^ for closed-canopy forest and 0.6 individuals /km^2^ in secondary forest. The encounter rate detection is relatively similar in both closed-canopy forest and secondary forest in the region. Calabar angwantibo, despite being one of the nocturnal primate species with limited data in the region, represents the uniqueness of the area as an epicenter of species richness and conservation. The information presented here represents current species update and underlines the significance of species monitoring, which provides valuable insight into the cryptic nocturnal Calabar angwantibo population in the Oban Hills Region rainforest ecosystem.

## Figures and Tables

**Table 1:** The density, abundance estimated, and encountered rate of the Calabar angwantibos (*Arctocebus calabarensis*) in Oban Region Hills of CRNP.

Vegetation Types	*n*	*k*	L	N	ER	AIC	ΔAIC	ESW (m)	Density/km^2^ (CI)	SE	(GOF) K-S	*p*
Closed-canopy forest	19	16	361	6515	0.52	97.36	−1.93	8.33	3.15 (1.75–5.69)	0.11	0.49	*0.59*
Secondary forest	22	16	367	830	0.63	118.05	−1.90	13.22	2.26 (1.20–4.26)	0.23	0.38	0.94
Pooled Estimates	41	32	728	7345	2.58	215.41	−3.83	21.55	2.57 (1.52–4.37)	0.34	0.87	1.35
Global Estimates	41	32	1227	4456	0.33	215.74	−1.66	10.68	1.56 (0.97–2.51)	0.12	0.46	0.76

Note: number of sightings (n), density (D/km^2^), 95% confidence interval (CI), survey efforts (L), abundance (N), encounter rate (ER), effective stripe width (ESW), Akaike information criterion (AIC), delta-AIC (ΔAIC), standard error (SE), number of sample/transects (*k*), detection probability (*p*)*,* Kolmogorov–Smirnov goodness of fit test (GOF) K-S.

## Data Availability

The data presented in this study are available on request from the corresponding author. The data are not publicly available due to threatened (endangered) status of the species.
